# Real-time monitoring of bacterial growth kinetics in suspensions using laser speckle imaging

**DOI:** 10.1038/s41598-019-57281-2

**Published:** 2020-01-15

**Authors:** Hadi Loutfi, Fabrice Pellen, Bernard Le Jeune, Roger Lteif, Mireille Kallassy, Guy Le Brun, Marie Abboud

**Affiliations:** 1 Physics Department, UR TVA, Faculty of Science, Saint Joseph University, B.P. 11-514, Riad El Solh Beirut, 1107 2050 Lebanon; 2 Laboratoire OPTIMAG, IBSAM, Université de Bretagne Occidentale, 6 avenue Le Gorgeu, C.S. 93837, 29238, Brest Cedex, 3 France; 3 Chemistry Department, UR TVA, Faculty of Science, Saint Joseph University, B.P. 11-514, Riad El Solh Beirut, 1107 2050 Lebanon; 4 Faculty of Science, Biotechnology Laboratory, UR EGP, Saint Joseph University, B.P. 11-514, Riad El Solh Beirut, 1107 2050 Lebanon

**Keywords:** Applied optics, Biological physics, Microbiology

## Abstract

In microbiology, monitoring the growth of any microorganism in culture is important for studying and optimizing the growth kinetics, the biomass and the metabolite production. In this work, we show that laser speckle imaging is a reliable technique that can be used to perform real-time monitoring of bacteria growth kinetic in liquid culture media. Speckle parameters, specifically speckle grain size and the spatial contrast of the speckle images, and standard analytical parameters (optical density, pH and colony forming units) were measured during the culture of different strains of *Bacillus thuringiensis*. Our results show that both speckle grain size and spatial contrast decrease with bacterial growth. Furthermore, speckle parameters are sensitive to the fermentation conditions. Statistical analysis revealed a relatively high correlation between speckle and analytical parameters.

## Introduction

Studying the growth of any microorganism in culture is crucial for understanding its growth kinetics and improving its yield. Once microorganisms are inoculated in a given culture medium, parameters such as the cell number counting^1^, oxygen consumption^2^, the pH of the culture medium^3^ or the optical density^4^ can be used as standard analytical parameters to monitor microorganism growth.

Other different techniques were developed as well for evaluating bacterial growth kinetics. Citing a few, the impedance technique, for example, can detect the effect of temperature on *Salmonella enteritidis* growth^5^. In addition, the colorimetric sensing array method allows identifying the strain or species of bacteria grown on agar plates using a colorimetric sensor^6^. Although these methods are reliable, optical techniques have become the preferred ones, as they are non-destructive. It was established that it is possible to identify different bacterial strains using the Mueller matrix imaging method^7^. Moreover, the effect of humidity on the growth of *Bacillus thuringiensis* was studied using an automated scanning microscope^8^. Despite the multiple advantages offered by these methods for monitoring bacterial growth kinetics, their disadvantage is that they are relatively expensive or they require lengthy results processing; furthermore, they need highly qualified personnel. To overcome these drawbacks, the biospeckle, an optical method based on the scattered light, has been extensively implemented in different fields^9–11^, particularly for characterizing fermentation products^12^ or investigating bacterial colony growth^13–16^. Reported studies were all performed on bacterial colonies growing on solid media (e.g., on an agar plate). However, since most of the culture of microorganisms at an industrial scale is conducted in bioreactors or fermentation tanks containing a liquid medium, it would be interesting to use biospeckle to monitor the growth kinetics of microorganisms in such media.

We used *Bacillus thuringiensis* as a model bacterium to demonstrate the efficiency of biospeckle for monitoring bacterial growth. *Bacillus thuringiensis* (commonly called Bt) is a Gram-positive, spore-forming bacterium that synthesizes parasporal crystals during the sporulation phase of its growth cycle. One of the main uses of this bacterium is for the biological control against insect pests. Among the entomopathogenic bacteria, Bt is both the most widely used species and the one that offers the best insecticidal potential for plant protection. It accounts for 90% of the microbial insecticide world market^17^. Commercial formulations of Bt consist of spore and crystal mixture^18^. When nutriments in the culture medium become limited, Bt enters a differentiation process, resulting in the formation of specialized form of cells, the spores. The later are more resistant to physical and chemical agents, and allow the bacteria to survive in a dormant state. During sporulation, Bt also synthesizes proteins, called δ-endotoxins (or Cry proteins), which accumulate in the mother cells as parasporal inclusions or crystals^19^. These inclusions of crystalized protein, representing up to 25% of bacterial dry weight, are the direct responsible for the larvicidal activity of Bt^20^. Different strains of Bt produce different insecticidal crystals (in terms of geometric shape, δ-endotoxins composition and structure), each specific to fight against a target pest: lepidopteran, dipteran, and coleopteran^21^. δ-endotoxins act on the cells of the mid-gut intestinal epithelium. The crystals ingested by the insect larvae are first solubilized in their intestinal cavity. The active fragment of δ-endotoxins is then released by the intestinal proteases of the susceptible insects. Indeed, only activated δ-endotoxins can cross the peritrophic membrane, which only allows small molecules to pass. The later will then bind to specific receptors on the surface of the epithelial cells. The toxin then induces pore formation in the membrane of the epithelial cells, resulting in rapid and almost total destruction of the intestinal epithelium. At the physiological level, intoxication manifests itself by an almost immediate paralysis of the digestive tract, which leads to a cessation of food intake. This allows spores that have been ingested at the same time as the crystals to germinate, and bacteria from this germination to multiply in the insect, causing sepsis^22^.

In our work, we demonstrate that biospeckle is a powerful alternative method for real-time monitoring of the kinetic growth of *Bacillus thuringiensis*. Four different strains of Bt were cultured in a liquid medium. Biospeckle parameters such as the speckle grain size^23^ and the spatial contrast^24^ were analyzed. Principal component analysis indicated a high correlation between speckle results and analytical parameters (optical density: OD, culture medium pH and colony forming units: CFU), and hence validates the capacity of speckle for monitoring the kinetic growth of bacterial concentration in a liquid culture.

## Results

### The biospeckle phenomenon

**Figure 1:**
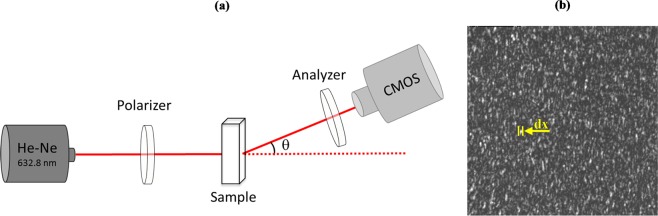
The speckle experimental setup. (**a**) is a diagram of the setup. The polarizer is used in order to create a linear polarization. The analyzer is used to select the parallel linear polarization. The sample contains 2 mL of cellular culture. The distance between the sample and the camera is fixed at 25 cm and θ is equal to 20°. (**b**) is an example of recorded speckle image where we show the horizontal size *dx* of the speckle grain.

The speckle phenomenon occurs when coherent light is scattered by an illuminated surface or scattering medium. The scattered waves, having random amplitudes and phases, arriving at any meeting point (a camera in our case), will interfere with each other generating a random intensity distribution called the speckle field^25^. Particles suspended in a liquid medium are subject to Brownian motion dynamics, which affect phase relationships and, consequently, the speckle image. When scatterers concentration in the sample increases, the dynamics of Brownian motion also increase and the speckle image then carries this information. The experimental set-up illustrated in Fig. 1(a) was used to follow the progression of different Bt strains growth in a liquid culture medium throughout fermentation time. Given that the geometrical dimension of the bacteria is larger than the optical wavelength (Bt are rod-shaped bacteria with an average length of 4.5 μm^26^), we assumed that a Mie scattering regime would occur, favoring forward scattering and thus we put the experimental setup in transmission^27^. Incident light interacts with a sample of culture medium containing bacteria. A CMOS camera then collects scattered light. The speckle image shown in Fig. 1(b) is the result of interference between scattered wavelets, where the dark spots represent a destructive interference and the bright spots (called the speckle grain) correspond to a constructive interference^28^.

We used four different strains of *Bacillus thuringiensis* in our experiment: two Bt var. *kurstaki* strains (HD-1 and LIP^MKA^) and two Bt var. *israelensis* (AM65–52 and AR23). The *kurstaki* strains produce bipyramidal and cubic crystals that can be used in plant protection against lepidopteran larvae^29^ and the *israelensis* strains synthesize spherical crystals used against dipteran larvae^30^. The *kurstaki* HD-1 strain and the *israelensis* AM65−52 strain were considered as reference strains since they have been widely studied and exploited over many years in research, whereas LIP^MKA^ and AR23 are strains collected and isolated from Lebanese soil^29^. T3 culture medium was used because its composition promotes the sporulation of the Bt cells^29,31^. Our work therefore had a double objective. On the one hand, it aimed to prove that the speckle imaging technique is a reliable and robust method for monitoring bacterial concentration in liquid media (regardless of the strain in question). On the other hand, it aimed to compare the growth kinetics of the reference strains with the Lebanese ones.

During measurements, optical density OD (considered as log(OD)) of the culture medium, its pH, and colony forming units were used as analytical reference parameters.

### A direct tool for monitoring the growth of Bt cell concentration

#### Speckle grain size

**Figure 2:**
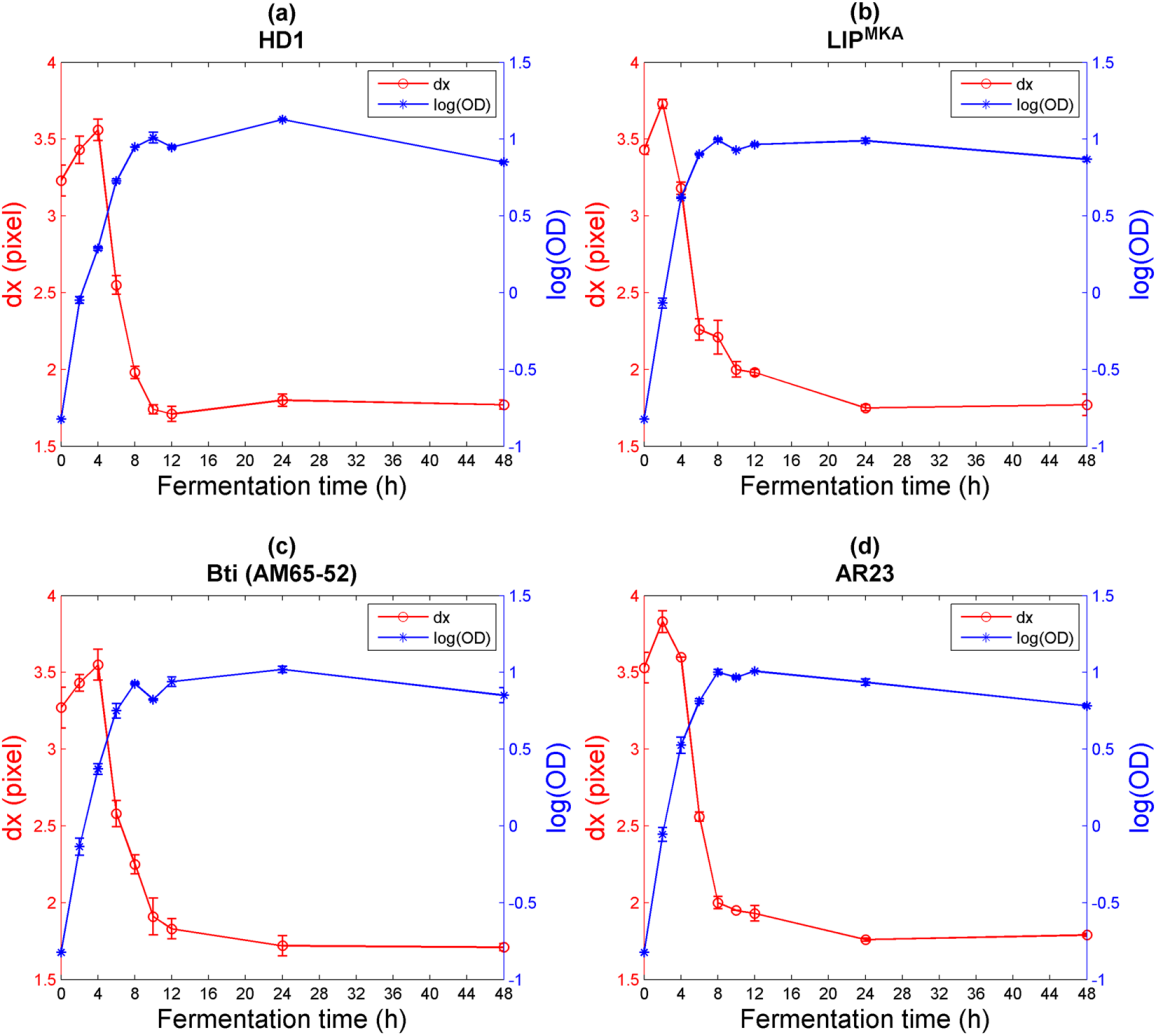
Variation of the speckle grain size *dx* and the log of the optical density OD as a function of fermentation time for the four strains (**a**) HD1, (**b**) LIP^MKA^, (**c**) Bti (AM65−52), and (**d**) AR23. Error bars correspond to the standard deviation resulting from three different experiments.

When considering second-order statistics on speckle images, one can study the speckle grain size *dx* related to the number or the size of the scatterers in the medium^32^. The evolution of *dx* as a function of fermentation time in the cultures of our study is presented in Fig. 2(a–d).

The four different strains of Bt show similar variations of *dx* as a function of fermentation time. This similarity demonstrates the standardization of the culture medium to monitor bacterial concentration over time and provides an optical signature for Bt cells. The decrease of *dx* could be directly linked to the augmentation of the bacterial concentration in the medium throughout the kinetic growth process. Indeed, as long as fermentation time continues, the bacterial number in the culture medium increases. The later increases the concentration of Mie scatterers and, therefore, an expansion of the diameter of the scattering spot. The speckle grain size *dx* will thus decrease, as predicted by Li and Chiang^33^.

Furthermore, speckle grain size *dx* can be used to compare bacterial growth efficiency among different strains. For the reference strains (HD-1 and Bti (AM65–52)), the decrease of *dx* began 4 hours after inoculation. However, for the Lebanese strains (LIP^MKA^ and AR23), the peak of the curve appeared only 2 hours after inoculation. This may indicate that the Lebanese strains start their exponential growth earlier than the reference strains in the T3 culture medium over the first 12 hours. Values of log(OD) for the four strains support this hypothesis: For the *kurstaki* strains, HD-1 had a log(OD) value of 0.288 after 4 hours of fermentation, while the LIP^MKA^ strain attained a log(OD) value of 0.618 after the same fermentation duration. In addition, for the *israelensis* strains, the AM65–52, the reference strain, had a log(OD) value of 0.370, however, the AR23 had a log(OD) value of 0.525, indicating a higher concentration of bacteria in the Lebanese strains.

The value of the angle between the laser-sample axis and the sample-CMOS camera axis was set at 20° in order to adapt the experimental setup to the wide dynamic range of the scattering process during bacterial growth and to ensure that only scattered photons were detected. When looking deeper at the first values of *dx*, we notice a slight increase. This may be because, at the beginning of fermentation, only the central area of the laser diffusion spot contributes principally to the diffusion phenomenon. Additionally, based on the optical density measurements, the dynamic growth of Bt bacteria can be seen to increase rapidly. Therefore, the angle of 20° ensures that only scattered photons are detected. Hence, the first points correspond to a simple diffusion regime that dominates before the fermentation process begins. After that, a transition to a multi-scattering regime takes place and *dx* starts decreasing as the fermentation process continues.

#### Speckle spatial contrast

**Figure 3:**
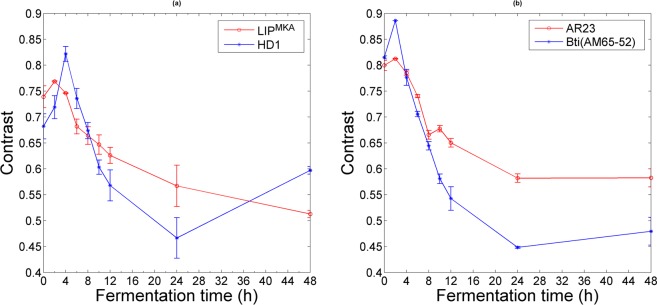
Variation of the spatial contrast as a function of fermentation time. (**a**) and (**b**) correspond to the two strains LIP^MKA^ and HD1 that form bipyramid and cubic crystals, and to the strains AR23 and Bti (AM65–52) that synthetize spherical crystals, respectively. Error bars correspond to the standard deviation resulting from three different experiments.

In addition to the speckle grain size *dx*, the spatial contrast of the speckle image can be used to follow the growth of Bt cells in a liquid culture medium. In general, this parameter provides information about the nature of the medium, for example its viscosity^10^ or the number of diffusers^34^. Moreover, the contrast of the image can detect the dynamics in the medium due to variation of the speckle grains. As shown in Fig. 3(a,b), spatial contrast decreases as the fermentation goes on and therefore as the concentration of scatterers in the medium increases.

Parameters that should be taken into account when discussing the variation in spatial contrast include the exposure time of the camera, the pixel size of the detector, the detected signal level and the dynamics of the scattering particles in the medium (see Eq. 8). When considering several values of camera exposure time (0.25 and 0.5 ms), we observed a similar trend to the speckle grain size^35^. Consequently, the decline in contrast can be linked to the variation of the ratio of the speckle grain size and the pixel size of the camera. We deduced that this parameter could be indirectly connected to an increase in Bt cell concentration during fermentation.

### Correlation between speckle and analytical parameters

In order to reveal potential correlations between speckle and analytical parameters, we performed a statistical analysis using principal component analysis (PCA) with XLSTAT. PCA allows us to find relationship between the parameters and builds a connection between them^36^. In our analysis, we took into account results of the analytical methods (optical density: OD; Colony Forming Units: CFU (measured as Viable Spores: VS); pH of the culture medium) and speckle parameters (speckle grain size: *dx* and spatial contrast: C). Results in Table 1 show the correlation between the variables. The best correlated results are *dx* and OD, with a Pearson correlation coefficient of −0.896. The negative value simply indicates that speckle and biological parameters vary in opposite ways. Both parameters are a direct measure of all kinds of scatterers present in the medium.

**Table 1:** Pearson correlation coefficient matrix between values of speckle parameters and analytical parameters for the growth kinetics of Bt bacteria for the four strains.

Variables	OD	pH	CFU(VS)	*dx*	Contrast
OD	1	—	—	—	—
pH	0.684	1	—	—	—
CFU(VS)	0.377	0.838	1	—	—
*dx*	−0.896	−0.840	−0.494	1	—
Contrast	−0.765	−0.868	−0.725	0.850	1

A correlation was also found between the pH and CFU(VS) (correlation coefficient = 0.838). This explains the increase in pH with the increase in the number of the bacterial cells throughout the growth of the Bt. In addition, the spatial contrast was highly correlated with all other parameters. However, this high correlation cannot be easily understood since the values of spatial contrast do not depend only on the dynamic and the characteristics of the culture but also on several parameters acquisition, such as the signal to noise ratio, speckle grain size and exposure time, as seen previously. Finally, we see that the CFU(VS) and *dx* are poorly correlated (a correlation coefficient of −0.494). Indeed, CFU(VS) is related directly to the sporulation dynamics. However, unlike CFU(VS), *dx* is sensitive to the total increase in the number of scatterers generated by the fermentation process.

**Figure 4:**
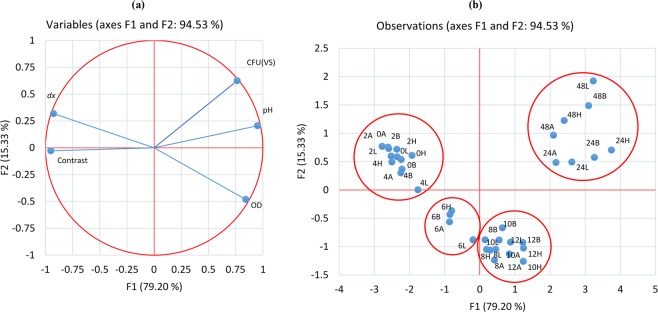
Representation of principal component analysis data (**a**) Pearson correlation circle representing the variables projected on the first (F1) and second (F2) principal components for the four Bt stains. (**b)** Observations grouped according to the fermentation time for the 4 Bt strains. The initials H, L, B and A correspond to the strain names: H for HD1, L for LIP^MKA^, B for Bti (AM65–52) and A for AR23 strains respectively. The numbers indicate the fermentation time in hours.

The Pearson correlation circle (Fig. 4(a)) features two principal components F1 and F2, representing 79.20% and 15.33% of the results, respectively. The F1 and F2 interrelation is equal to 94.53%. Moreover, the Pearson circle authenticates the notion of variation in opposite directions for speckle grain size *dx* and concentration of Bt cells mentioned above. This analysis is also in good agreement with the Li and Chiang formula which explains that when the bacterial concentration increases, the diameter of the scattering spot will also expand, resulting in a smaller speckle grain size^33^. Figure 4(b) shows a graph representing the observation variables. One can clearly distinguish four phases that can be attributed to the four phases of the Bt fermentation process^37^. Phase I, known as the vegetative growth phase, includes the points related to 0, 2, and 4 hours and means that the exponential growth of vegetative cells takes on average 4 hours. Phase II contains the 6-hour point and it corresponds to the deceleration phase of the growth process. Phase III of the process includes the points at 8, 10, and 12 hours. This phase is highlighted by a variation in the growth curve (Fig. 2). It corresponds to the entrance to the stationary phase. Finally, the fourth phase in the fourth quadrant includes the points at 24 and 48 hours, and corresponds to the sporulation and the cell lysis.

**Figure 5:**
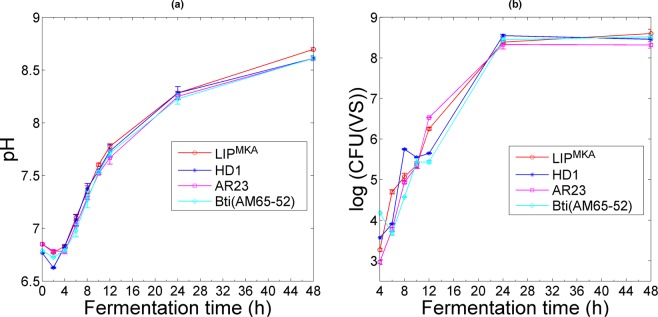
Variation of analytical parameters for the four strains as a function of fermentation time. (**a**) and (**b**) correspond to pH and log(CFU(VS)) respectively. For (**b**), the measurement of CFU(VS) was started at 4 hours because the bacterial cells did not sporulate during the first 2 hours. Error bars correspond to the standard deviation resulting from three different experiments.

Although the PCA shows high correlation values between speckle and analytical parameters (except *dx* and CFU(VS)), we should take a closer look at each analytical parameter to consider its limitations and to see how speckle can provide additional information. First, one can consider that the pH of the culture medium is a good indicator of its standardization (Fig. 5(a))^38^. Indeed, the nutrients sources in T3 medium are the yeast extract, the tryptose and the tryptone. We can see that for the first two hours, pH values decrease as the fermentation is in its early process; this is due to the organic acids production in the culture through sugar consumption. Afterward, as the Bt cells grew, they oxidize the organic acids that increased the pH of the culture, while developing, duplicating and sporulating^39^. This figure shows that the four graphs of pH, for the four different strains, are identical and overlapping, giving no detailed information about the number of bacterial cells in the batches or the differentiation between strains. Hence, pH cannot be considered as a key parameter while monitoring the dynamics of bacterial growth. Secondly, despite the fact that assessment of Colony Forming Units (CFU(VS)) is a very accurate method for monitoring the concentration of spores as shown in Fig. 5(b) (same layout as the log(OD)), the determination of the number of viable spores is very time-consuming. Finally, from an optical point of view, the complexity of the measurement of the optical density (OD) relies on the configuration of the spectrophotometer. The photosensor collects not only the transmitted light (or ballistic photons) but also the scattered light coming from the particles in the sample container, especially when diffusion reaches a high level (for example, when the concentration of the particles increases). In addition, OD measurements vary among spectrophotometers. Each apparatus has its own linear range. Thus, we cannot conclude that the optical density method is unreliable, but it requires a number of crucial considerations that should be addressed while monitoring bacterial growth kinetics. The speckle method seems to overcome all the described difficulties encountered in the other methods. First, speckle grain size *dx* (as shown above in Fig. 2) differs for the four strains, especially in the first 4 hours of fermentation, suggesting that each strain has its own growth dynamic. Secondly, speckle technique can rapidly provide the information about variation in concentration via speckle image processing (see Supplementary Figs. S1 and S2). Finally, yet importantly, scattered light coming from the diffusing particles is only taken into account by the speckle pattern, so dynamic speckle can provide a good estimation of the number and the size of optical scatterers.

### Effect of culture conditions on speckle grain size

**Figure 6:**
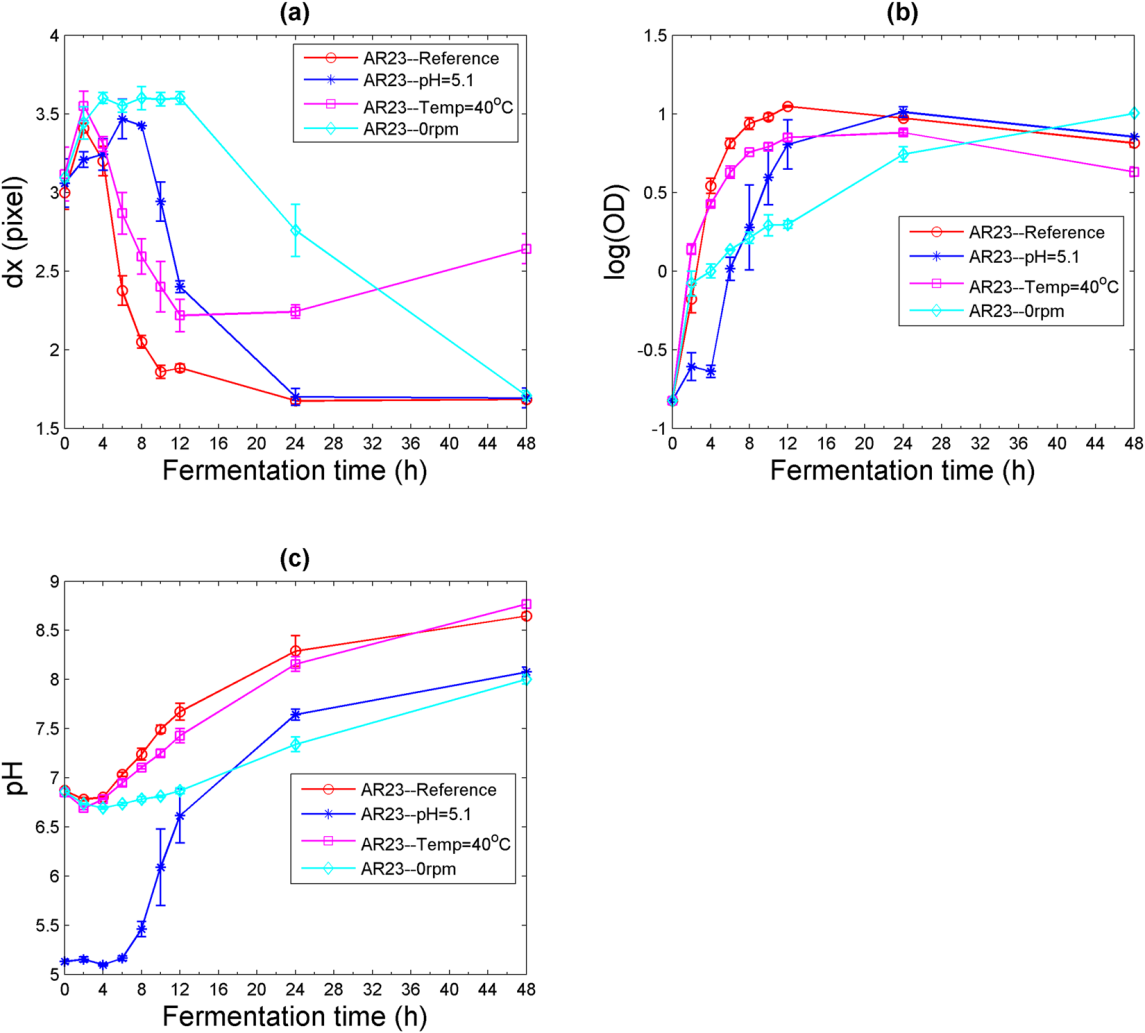
Variation of the speckle and culture parameters for the strain AR23 in four different growth conditions. (**a**) Speckle grain size *dx*, (**b**) log(OD), and (**c**) pH of the culture medium.

To demonstrate the sensitivity of the speckle imaging technique for monitoring the fermentation yield of the bacteria, we separately varied the growing conditions (pH, temperature and oxygenation) of the culture medium and considered their effects on speckle parameters. In fact, when a condition in the fermentation process is changed, the growth of the bacteria is affected and the production will yield a different quantity and quality of cells and metabolites. Four Erlenmeyers were prepared, each containing the culture medium and the inoculated AR23 strain. For each Erlenmeyer, one of the growth conditions was modified. The first Erlenmeyer was considered as the control, with optimal conditions (temperature = 30 °C, pH = 6.8 and oxygenation = 250 rpm). For the second Erlenmeyer, the pH was modified to 5.1, while keeping the temperature at 30 °C and the oxygenation at 250 rpm. This acid pH would decelerate the fermentation process at its early stage without preventing the sporulation of the Bt cells^40^. The temperature was increased to 40 °C for the third Erlenmeyer in order to thermally stress the bacteria, while pH was maintained at 6.8 and oxygenation rate at 250 rpm^41^. Finally, for the fourth Erlenmeyer, the shaker in the incubator was turned off (0 rpm), while the temperature and the pH were kept the same as for the control sample^42^. Each Erlenmeyer was prepared in triplicate and mean values with standard deviations are given in Fig. 6. Although similar general trends were obtained with the analytical parameters, significant differences are observed in *dx* when any of the culture conditions is changed. As shown in Fig. 6(a), the speckle grain size *dx* can be a significant tool to determine if we have good cell production, especially during the first twelve hours of the fermentation process. Based on the *dx* curves, we can note that, among the experimental conditions tested, temperature had the least effect on bacterial growth via the fermentation process. Oxygenation, however, was shown to be an essential element for a good fermentation yield since at 0 rpm a plateau occurred in the *dx* curve in the first 12 hours of fermentation time^43^. The graph corresponding to the variation of log(OD) and pH as a function of fermentation time (Fig. 6(b,c)) shows there was a large difference in cell production between the four different conditions over the first twelve hours. One can thus use speckle imaging analysis as a qualitative and quantitative test for the estimation of the fermentation yield in laboratories or industries and the effect of fermentation conditions on this yield.

## Discussion

In this paper, the growth kinetics of four different strains of *Bacillus thuringiensis* (Bt), LIP^MKA^, HD-1, AR23 and Bti (AM65–52) were monitored using the speckle imaging technique. Experimental and statistical results show a negative and yet high correlation between speckle parameters (speckle grain size *dx* and spatial contrast C) on one hand and the concentration of Bt bacteria in a culture medium on the other. One of the main advantages of the speckle method is the variation in speckle grain size with the concentration of Bt cells. The peak observed in the *dx* curves shows the Phase I of the Bt fermentation process, while this phase is totally covered by the other analytical parameters. This reveals that the speckle grain size is very sensitive to the rapid variations in growth kinetic, especially in the early stages of the fermentation, because at the beginning of the process, we have a simple diffusion of light and after a few hours, the multi-diffusion regime takes place due to the adequate choice of the detection angle. Moreover, by changing the culture conditions, we showed that speckle imaging is efficient for tracking the growth of Bt concentration. A strong correlation was found between speckle and analytical parameters, with the Pearson circle showing an anti-correlation between these parameters. Furthermore, statistical analysis with PCA revealed four distinct phases during fermentation. Finally, one can further develop the setup by implementing an *in vitro* device that allows a real-time monitoring of the fermentation without any sampling, in opposition to the standard analytical methods where a sampling of the culture medium is required. Our results therefore establish for the first time, to the best of our knowledge, that speckle is a rapid since less than 6 seconds is required to obtain the result, non-invasive, and yet valid method for monitoring bacterial growth kinetics in suspension. It thus provides an alternative method to the standard approaches used by microbiologists to follow the kinetic growth of Bt cells and, more widely, of any bacterial cultures in liquid media. Indirectly, this method can also be used to detect contamination in clear culture media before use: it can be used to measure turbidity variation in culture media and thus reveal the presence of any contaminating bacteria.

## Materials and Methods

### Sample handling

Two Bt var. *kurstaki* strains were considered: HD-1, as a reference strain, and LIP^MKA^, a strain extracted and isolated from Lebanese soil, synthesizing bipyramidal and cubic δ-endotoxin crystals. In addition, two Bt var. *israelensis* strains were also used: AM65–52 (provided by Christiane Nelsen LeRoux from MICALIS institute INRA Jouy-en-Josas), used as a reference strain, and AR23, strain extracted and isolated from Lebanese soil both, synthesizing spherical crystals. The four strains were initially maintained on an agar LB medium (Luria-Bertani Broth) at 4 °C. For the pre-inoculum, the strains were inoculated in 500-mL Erlenmeyers containing 100 mL of LB medium and incubated in a rotary shaker overnight (between 14 and 16 hours). For the inoculum preparation, T3 liquid medium was used, containing: Tryptose 2 g.L^−1^, Tryptone 3 g.L^−1^, yeast extract 1.5 g.L^−1^, Na_2_HPO_4_ 1.5 g.L^−1^, NaH_2_PO_4_ 1.1 g.L^−1^, MnSO_4_, MgSO_4_ solution 2 mL, and ionic solution 1 mL (containing in 100 mL of H_2_O: ZnCl_2_ 3.407 g, MgCl_2_.6H_2_O 50.825 g, MnCl_2_.4H_2_O 0.989 g, CaCl_2_ 11.098 g, FeCl_3_ 4.080 g)^29^. Operating conditions were as follows: a volume of 150 mL of T3 liquid medium in a 1-L Erlenmeyer, temperature set at 30 °C, pH at 6.8 and a stirrer speed of 250 rpm. At the beginning of our study, the culture medium was inoculated with a number of Bt cells corresponding to an optical density of 0.15.

### Speckle experimental setup

The speckle experimental setup is drawn in Fig. 1(a). A linearly polarized 15-mW He-Ne laser (HNL150LB, Thorlabs), operating at 632 nm where the absorption of the medium is minimal, illuminates a quartz cell (101-QS, Hellma) containing T3 culture medium with the different Bt cells. The light beam passes through a polarizer (LPVIS050, Thorlabs) before it reaches the sample. The sample is a quartz cell of 10 mm × 10 mm × 45 mm, containing 2 mL of the liquid culture medium with the Bt cells. Transmitted scattered light then goes through an analyzer (LPVIS050, Thorlabs) and is finally collected by a high-speed recording Complementary Metal Oxide Semiconductor CMOS camera (MotionBLITZ EoSens mini1, Mikrotron GmbH, pixel size 14 μm × 14 μm). The speckle images (Fig. 1(b)) are acquired using linear parallel (LP) light polarization configuration. The camera exposure time and framerate are taken equal to 0.5 ms and 1950 fps, respectively. We have set the time exposure value to 0.5 ms in all the experiments. The time exposure value corresponds to a compromise between the signal-to-noise ratio in speckle patterns and the Brownian motion of Bt cells in the culture medium. The distance between the sample and the camera was set at 25 cm and the angle θ between the camera and the optical axis at 20° in order to avoid direct beam detection and to ensure that only scattered photons were detected. In our experiment, we took 2 mL of the liquid culture medium with the Bt cells and imaged them using speckle setup. The acquisition of 1000 images takes less than 1 second. In addition, the image processing time is estimated to a maximum of 5 seconds. Therefore, a total of 6 seconds is required to obtain the result.

### Speckle extracted parameters

Spatial parameters were calculated in the analysis of speckle patterns. The average speckle grain size *dx* is calculated by the normalized auto-covariance function ${c}_{I}(x,y)$ of the speckle intensity pattern $I(x,y)$^25^. First of all, normalization of the autocorrelation function is an essential step to determine *dx*. So, if $I({x}_{1},{y}_{1})$ and $I({x}_{2},{y}_{2})$ are the intensities of two random points in the observation plane (*x,y*), the autocorrelation function *R*_*I*_ is defined by Eq. (1):

1 $${R}_{I}(\Delta x,\Delta y)=\langle I({x}_{1},{y}_{1})I({x}_{2},{y}_{2})\rangle $$

where $\Delta x={x}_{1}-{x}_{2}$, $\Delta y={y}_{1}-{y}_{2}$ and $\langle \rangle$ represent the spatial average between the two intensities. If ${x}_{2}=0$, and ${y}_{2}=0$, ${x}_{1}=x$ and ${y}_{1}=y$, we can write:

2 $${R}_{I}(\Delta x,\Delta y)={R}_{I}(x,y)$$

The auto-covariance function is given by:

3 $${c}_{I}(x,y)=\frac{{R}_{I}(x,y)-{\langle I(x,y)\rangle }^{2}}{\langle I{(x,y)}^{2}\rangle -{\langle I(x,y)\rangle }^{2}}$$

Based on the Wiener-Khintchine theorem^44^, we can find the autocorrelation function ${R}_{I}(x,y)$ by applying the Inverse Fourier Transform on the Power Spectral Density (PSD) of the intensity of the speckle image:

4 $$PS{D}_{I}(x,y)={|FT[I(x,y)]|}^{2}$$

Thus:

5 $${R}_{I}(x,y)=F{T}^{-1}[PS{D}_{I}(x,y)]$$

Mixing Eqs. (4) and (5) and replacing it in Eq. (3), we can obtain the auto-covariance function:

6 $${c}_{I}=\frac{F{T}^{-1}[{|FT[I(x,y)]|}^{2}]-{\langle I(x,y)\rangle }^{2}}{\langle I{(x,y)}^{2}\rangle -{\langle I(x,y)\rangle }^{2}}$$

We estimated *dx* by the width at half maximum of a horizontal cut taken from the speckle image auto-covariance function (see Supplementary Fig. S2).

Speckle grain size *dx* can also be estimated using the Li and Chiang equation^33^:

7 $${\rm{d}}{\rm{x}}{\rm{=}}\frac{{\rm{1.22}}\times {\rm{D}}\times \lambda }{{{\rm{D}}}_{{\rm{e}}}\times \,\cos \,{\theta }}$$

where *D*, λ, *De* and *θ* are the distance between the sample and the camera, the wavelength of the laser, the diameter of the scattering spot and the angle between the incident beam and the one detected by the camera, respectively.

Another parameter used in our study is the spatial contrast of the speckle image^45^, given by:

8 $$C=\frac{\sigma }{\langle I\rangle }$$

where $\langle I\rangle$ is the speckle image mean intensity and σ the standard deviation of the gray level intensity. C values range between 0 and 1.

### Standard analytical analysis

Standard biological measurements were performed concomitantly on the different Bt strains, while studying the kinetic growth of the later. pH was measured throughout the fermentation process and optical density (OD) was estimated using a spectrophotometer (Purkin Elmer, MBA 2000) at a wavelength of 600 nm, with a dilution factor that allows measurements in the linear range. Samples were also analyzed for the number of colony forming units (CFU) of heat resistant spores (VS) by the serial dilution plating technique^46^. The appropriately diluted samples were plated and incubated overnight at 30 °C to form fully developed colonies. CFU (VS/mL) provides information about the concentration of spores in the batch.

## Supplementary information

Supplementary Information.
