# Supplementary material for: Real-time monitoring of bacterial growth kinetics in suspensions using laser speckle imaging
Source: Sci Rep. 2020 Jan 15;10:408. doi: 10.1038/s41598-019-57281-2 (PMC6962325; doi:10.1038/s41598-019-57281-2)
Supplement: Supplementary file 1 — Supplementary Information. [file 41598_2019_57281_MOESM1_ESM.pdf]

## Title

# Real-time monitoring of bacterial growth kinetics in suspensions using laser speckle imaging


### Authors

Hadi Loutfi[1, 2, *], Fabrice Pellen[2], Bernard Le Jeune[2], Roger Lteif[3], Mireille Kallassy[4], Guy Le Brun[2], Marie Abboud[1]

### Affiliations

[1] Physics Department, UR TVA, Faculty of Science, Saint Joseph University, B.P. 11-514 Riad El Solh Beirut 1107 2050, Lebanon, marie.abboud@usj.edu.lb

[2] Laboratoire OPTIMAG, IBSAM, Université de Bretagne Occidentale, 6 avenue Le Gorgeu, C.S. 93837, 29238, Brest Cedex 3, France, guy.lebrun@univ-brest.fr

[3] Chemistry Department, UR TVA, Faculty of Science, Saint Joseph University, B.P. 11-514 Riad El Solh Beirut 1107 2050, Lebanon

[4] Faculty of Science, Biotechnology Laboratory, UR EGP, Saint Joseph University, B.P. 11-514 Riad El Solh Beirut 1107 2050, Lebanon

* hadi.loutfi@net.usj.edu.lb, hadi.loutfi@etudiant.univ-brest.fr

## Supplementary Material

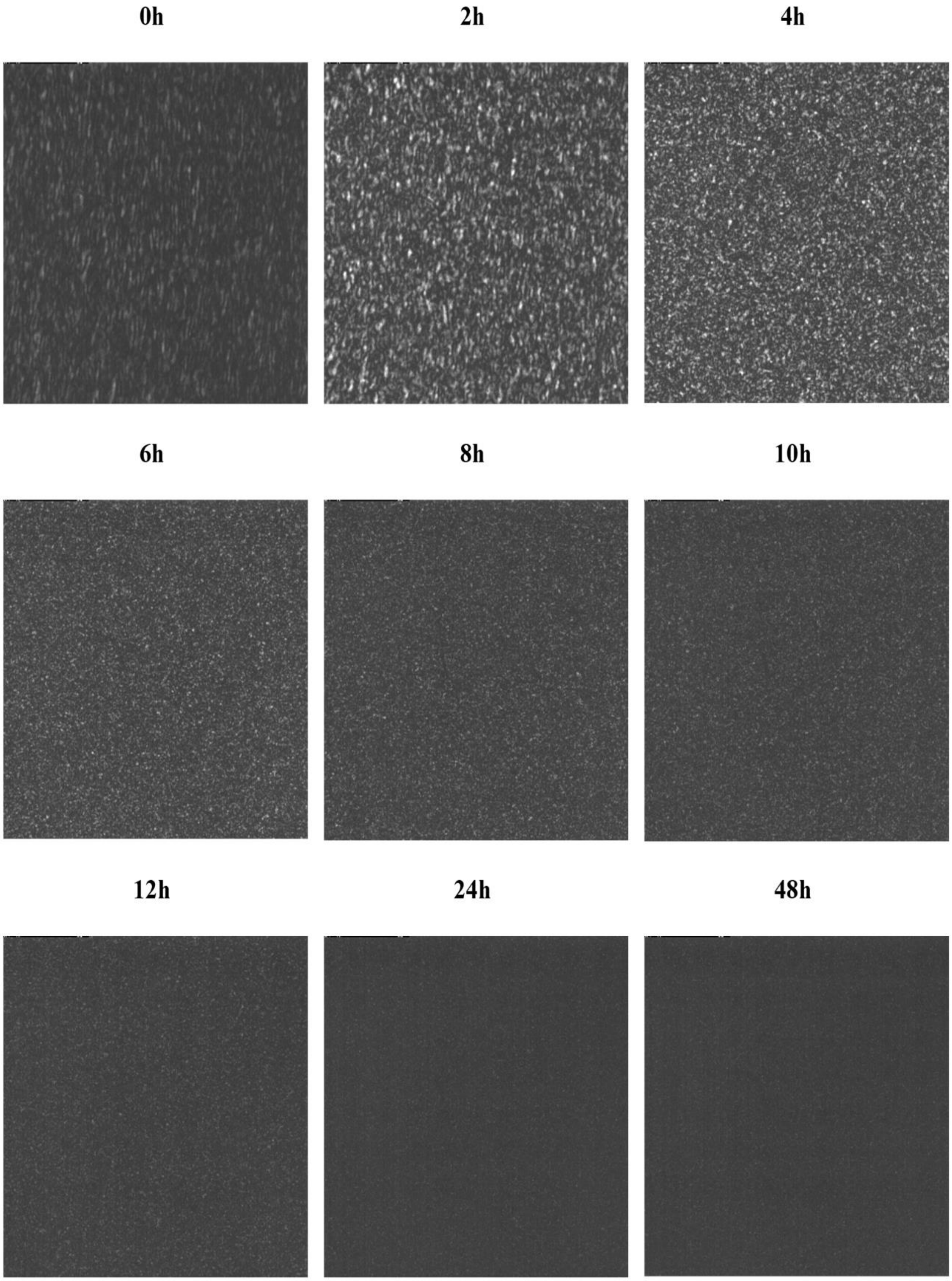


**Fig. S1 Variation of the speckle image with fermentation time for the LIP$^{MKA}$ strain**

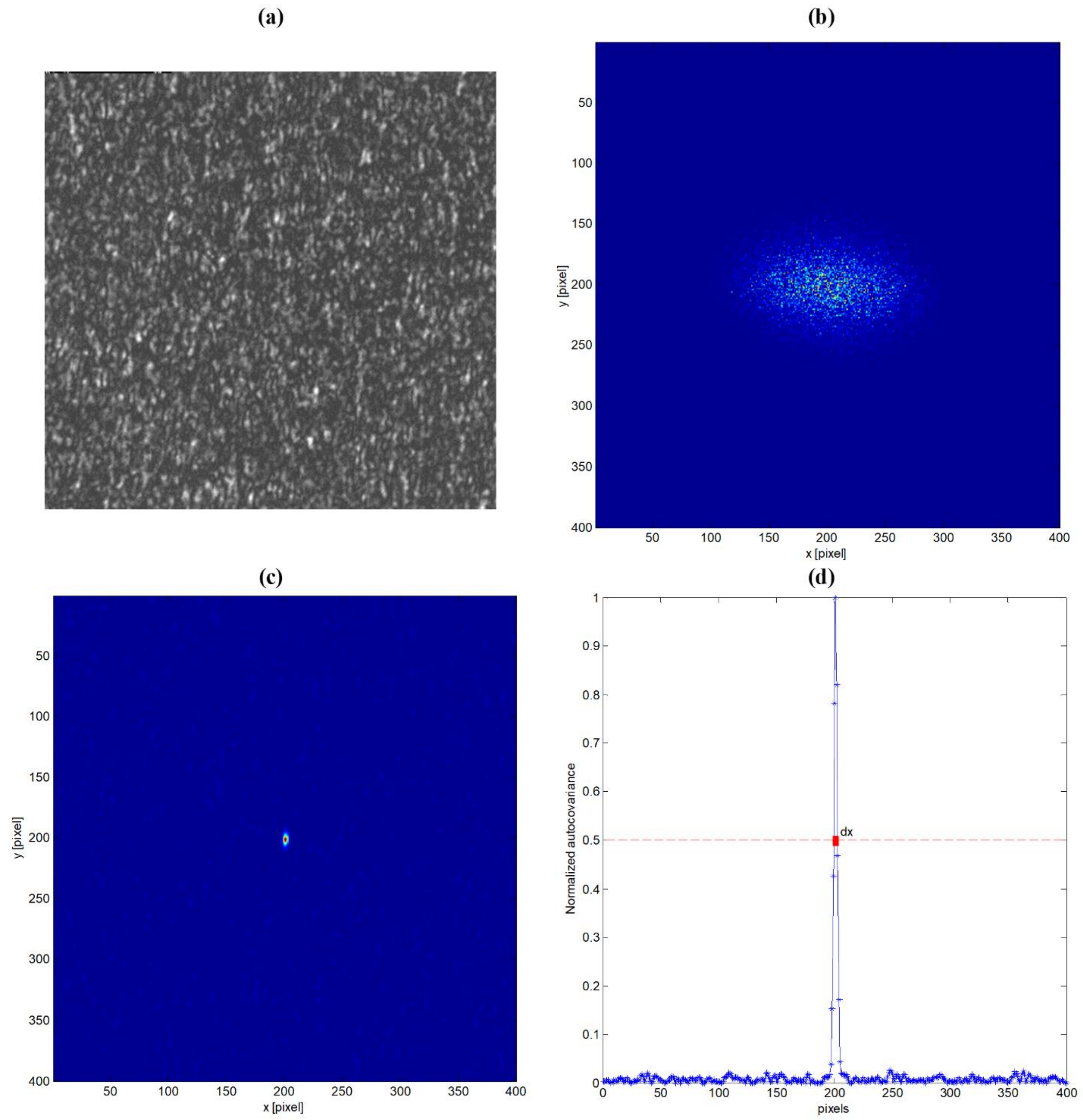


**Fig. S1 – Figures showing the procedure for the calculation of the speckle grain size. (a)** is an example of speckle image. **(b)** represents the Power Spectral Density (PSD) of the intensity of the speckle image given by Eq. (4). The low-frequency regions appear in the center of the image, while the high-frequency regions are located at the corners of the PSD image. **(c)** The autocorrelation function of the speckle figure is calculated by performing the inverse Fourier transform of the DSP (see Eq. (5)). **(d)** Finally, *dx* corresponds to the width at half maximum of a horizontal cut taken from the speckle image auto-covariance function illustrated by Eq. (6).
